# Characterization of DNA Polymerase Genes in Amazonian Amerindian Populations

**DOI:** 10.3390/genes14010053

**Published:** 2022-12-24

**Authors:** Amanda Cohen-Paes, Angélica Leite de Alcântara, Elisa de Souza Menezes, Fabiano Cordeiro Moreira, Marianne Rodrigues Fernandes, João Farias Guerreiro, Ândrea Ribeiro-Dos-Santos, Sidney Emanuel Batista Dos Santos, Ney Pereira Carneiro dos Santos

**Affiliations:** 1 Núcleo de Pesquisas em Oncologia, Universidade Federal do Pará, Belém 66073-000, PA, Brazil; 2 Laboratório de Genética Humana e Médica, Instituto de Ciências Biológicas, Universidade Federal do Pará, Belém 66075-110, PA, Brazil

**Keywords:** Indigenous, DNA polymerase genes, genetic variants, continental populations, Brazilian population

## Abstract

Due to their continuing geographic isolation, the Amerindian populations of the Brazilian Amazon present a different genetic profile when compared to other continental populations. Few studies have investigated genetic variants present in these populations, especially in the context of next-generation sequencing. Knowledge of the molecular profile of a population is one of the bases for inferences about human evolutionary history, in addition, it has the ability to assist in the validation of molecular biomarkers of susceptibility to complex and rare diseases, and in the improvement of specific precision medicine protocols applied to these populations and to populations with high Amerindian ancestry, such as Brazilians. DNA polymerases play essential roles in DNA replication, repair, recombination, or damage repair, and their influence on various clinical phenotypes has been demonstrated in the specialized literature. Thus, the aim of this study is to characterize the molecular profile of *POLA1*, *POLE*, *POLG*, *POLQ*, and *REV3L* genes in Amerindian populations from the Brazilian Amazon, comparing these findings with genomic data from five continental populations described in the gnomAD database, and with data from the Brazilian population described in ABraOM. We performed the whole exome sequencing (WES) of 63 Indigenous individuals. Our study described for the first time the allele frequency of 45 variants already described in the other continental populations, but never before described in the investigated Amerindian populations. Our results also describe eight unique variants of the investigated Amerindians populations, with predictions of moderate, modifier and high clinical impact. Our findings demonstrate the unique genetic profile of the Indigenous population of the Brazilian Amazon, reinforcing the need for further studies on these populations, and may contribute to the creation of public policies that optimize not only the quality of life of this population, but also of the Brazilian population.

## 1. Introduction

The human genome encodes important information to protect its integrity [1,2]. We know that DNA replication is carried out accurately and efficiently, essentially due to enzymes called DNA polymerases, which not only synthesize a daughter molecule via a DNA template molecule, but also have the ability to ”check their work” immediately after that synthesis, repairing base mismatches in a process called proofreading [2,3]. Despite this, the frequency of errors made by eukaryotic DNA polymerases is estimated to be approximately one error for every 10^4^–10^5^ nucleotides during each cellular S phase, partially because of environmental stressors such as ultraviolet sunlight, lifestyle habits such as smoking, or dietary factors [4,5]. Additionally, a large proportion of DNA changes are inevitably caused by endogenous mutagens, including reactive oxygen species and the action of metabolites in the organism [6]. Errors in DNA polymerases can lead to mutations or genomic alterations with the potential to cause cell lethality or lead to tumor formation and several other pathologies [7,8,9,10,11,12].

DNA polymerases play essential roles in DNA replication, repair, recombination, or damage repair, and their influence on various clinical phenotypes has been demonstrated in the specialized literature. Despite this, no research has reported the frequency or biological value of mutations in these genes in Amerindian populations. In fact, genetic studies in these populations are extremely rare, and to date their genomic data are unavailable in international and Brazilian project databases, such as the 1000 Genomes Project, The Exome Aggregation Consortium (ExAC), The Genome Aggregation Database (gnomAD), Arquivo Brasileiro Online de Mutações (ABraOM), and the Brazilian Initiative on Precision Medicine (BIPMed). Given that genomic ancestry is a determining factor in complex disease association studies, the unavailability of genomic data from Amazonian Indigenous populations limits the validation of previously consolidated data in other continental populations, besides compromising the clinical applicability of such findings and also the discovery and characterization of new markers unique to these populations [13,14,15]. The physical isolation of Brazilian Amerindians from urban centers is still a reality in several ethnic groups, so it is worth noting that even today there is a gap in the knowledge of epidemiological data of these individuals, with approximately 896,000 representatives distributed throughout the geographical territory of Brazil [16,17,18,19,20,21].

Additionally, recent studies have shown that Amerindian ancestry can modulate the predisposition to certain diseases, which means that knowledge of their molecular profile has a relevant impact not only for these populations, but also for admixed populations with strong Amerindian ancestry, as in the case of the Brazilian population [22,23,24,25,26]. Thus, the aim of this study is to characterize the molecular profile of *POLA1*, *POLE*, *POLG*, *POLQ*, and *REV3L* genes in Amerindian populations from the Brazilian Amazon, comparing these findings with genomic data from five continental populations described in the gnomAD database, and with data from the Brazilian population described in ABraOM.

## 2. Materials and Methods

### 2.1. Study Population and Ethics

The study was approved by the National Research Ethics Committee (CONEP; available at: http://conselho.saude.gov.br/comissoes-cns/conep/ accessed on 5 October 2022) and by the Research Ethics Committee of the Tropical Medicine Center of the Federal University of Pará (CAE: 20654313.6.0000.5172). Because we are dealing with Amerindian groups, the leaders of the investigated communities were contacted, and they signed an informed consent form for participation in the study.

A total of 63 Amerindians participated in the study, from 12 ethnic groups that occupy the geographical territory of the Brazilian Amazon: Asurini do Xingu; Asurini do Tocantins; Awa-Guajá; Arara/Arara do Iriri; Araweté; Kayapó/Xikrin; Karipuna; Zo’é; Wajãpi; Phurere; Munduruku and Yudjá/Juruna. For our analysis, we grouped the 12 ethnic groups mentioned above into a single population called the Native American Population (NAT).

For comparison analyses we also used data from 5 continental populations available in the Genome Aggregation Database (available at: https://gnomad.broadinstitute.org/; accessed on 15 February 2022). Thus, we included data from 8128 individuals from Africa (AFR); 56,885 from Europe (EUR non-finish); 17,296 from the Americas (AMR); 9197 from East Asia (EAS) and 15,308 from South Asia (SAS).

Additionally, we included the population described in the Brazilian Online Mutation Archive (ABraOM—available at: https://abraom.ib.usp.br; accessed on 5 October 2022), which represents the population of southeastern Brazil, specifically residing in the state of São Paulo.

### 2.2. Extraction of the DNA 

We collected peripheral blood from the individuals participating in the study. After that, DNA was extracted using the phenol-chloroform technique, described by Green and Sambrook et al. [27]. To quantify the genetic material extracted per sample, we used the Nanodrop-8000 spectrophotometer (Thermo Fisher Scientific Inc., Wilmington, DE, USA).

### 2.3. Bioinformatics Analysis

Whole Exome sequencing reactions were performed on the NextSeq 500^®^ platform (Illumina^®^, San Diego, CA, USA) using the NextSeq 500 High-output v2 Kit 300 cycle (Illumina^®^, San Diego, CA, USA).

Reads in FASTQ format were analyzed for quality using FastQC v.0.11 (http://www.bioinformatics.babraham.ac.uk/projects/fastqc/, accessed on 25 June 2021) in order to filter out and exclude all low-quality reads (below 10 reads) we used the tool Fastx_tools v. 0.13 (http://hannonlab.cshl.edu/fastx_toolkit/; accessed on 25 June 2021).

We aligned the sequences found with the GRCh38 genome, which is commonly used as a reference in next-generation sequencing studies. For the alignment, we used the BWA v.0.7 tool (http://bio-bwa.sourceforge.net/; accessed on 20 March 2022), and indexed the generated file (SAMtools v.1.2-http://sourceforge.net/projects/samtools/; accessed on 20 March 2022).

Picard Tools v.1.129 was used to remove duplicate reads (http://broadinstitute.github.io/picard; accessed on 20 March 2022) and GATK v.3.2 did the mapping quality recalibration and local realignment (https://www.broadinstitute.org/gatk; accessed on 20 March 2022). GATK v.3.2 (https://gatk.broadinstitute.org/hc/en-us; accessed on 20 March 2022) also performed the search for variants present in the 18 genes selected from the reference genome for analysis. Variant annotation was performed with the variant viewer software, Viva^®^ (Natal, RN, Brazil).

### 2.4. Statistical Analysis

The allele frequencies of the NAT population were obtained by counting in the population and calculating the frequency. The difference in frequencies between the investigated populations (NAT, ABraOM, FR, EUR, AMR, EAS and SAS) was analyzed by Fisher Test. All statistical analyzes were performed using the R Studio v.3.5.1 program (R Foundation for Statistical Computing, Vienna, Austria). The False Discovery Rate (FDR) was used to correct the multiple analyses.

### 2.5. Selection of Genetic Variants

We selected 5 component genes of the DNA replication and repair pathway in humans. The following selection criteria were applied to the exome sequencing result to perform the pairwise analysis: (I) the read should be high-coverage, with a minimum of 10 reads coverage; (II) the predicted impact should be “modifier”, ”moderate” or ”high” according to SNPeff (https://pcingola.github.io/SnpEff; accessed on 25 June 2021); (c) the difference in allelic frequency of the variants between NAT populations and continental populations should be significant (*p*-value ≤ 0.05). After applying selection criteria, 55 genetic variants were statistically analyzed.

## 3. Results

Our results identified 432 variants in total, with only 45 remaining after our selection criteria. Table 1 shows those 45 variants with their respective allele frequencies in the Amerindian, Continental and Brazilian populations. The chromosomal position, the mutant allele (variant), the wild allele (reference) and the gene are also described in the table. When analyzing the frequencies quantitatively, the Indigenous population (NAT) was more similar to the Latin American population (AMR) in most of the markers described. On the other hand, the African population (AFR) had the most different allele frequencies from the NAT population in the data below. Regarding the southeast Brazil population, represented by ABraOM data, 14 single nucleotide variants (SNV) stand out with significantly different frequency when compared to the Amazonian Amerindian population (rs4883537, rs4883538, rs4883543, rs5744750, rs4883544, rs4883613 and rs4883555, of the *POLE* gene; rs2072267l, rs2307433 and rs2307438 from *POLG* gene; rs3218636, rs3218651 and rs61757738 from *POLQ* gene; rs11376056 from *REV3L* gene). 

We emphasize that the frequencies of some variants identified in this work and attributed to the Indigenous population have never been calculated or described for other populations. Thus, Table 2 shows new and possibly unique variants for the Amerindian populations investigated. In the table, one can observe the eight new variants related to their respective gene, chromosomal position (Chrom) in the genome with detailed region, mutant allele (Variant), wild allele (Reference), the impact of the mutation, the type, changes it generates in protein formation and the type of change produced (Var type). Among the eight new variants, one is an insertion/deletion polymorphism (INDEL), with high impact, three are SNVs with modifier impact and four are missense SNVs with moderate impact.

Regarding the pairwise comparison of frequencies of the mutations described between NAT and each of the populations investigated (including the five continental populations and the southeastern Brazilian population described in ABraOM), we obtained a total of 14 variants that met our selection criteria. Table 3 shows where this analysis was statistically significant, such results are highlighted in bold. Thus, we can see that there was statistical difference for three variants of the POLE gene, two of which have allele frequency in the NAT population different from the European, South Asian, and southwestern Brazilian populations (rs4883544 and rs4883613), and one of them differs only from the EUR and ABraOM populations (rs5744761). For the *REV3L* gene, the rs462779 variant in the Indigenous population differed significantly from the AMR, SAS and ABraOM populations. Notably, INDEL rs3087377 of the *POLG* gene stood out among all the variants analyzed, as its frequency in the Amerindian population is statistically different from four of the five continental populations investigated in the 1000 Genomes database (AMR, EAS, EUR and SAS) and the Brazilian population of ABraOM (*p*–Value AMR = 0.0365; *p*–Value EAS = 0.0366; *p*–Value EUR = 0.0004; *p*–Value SAS = 0.0006; *p*–Value ABraOM = 0.058), thus being the marker that most demonstrated genetic differentiation of the population studied when compared to the others.

Regarding the distribution of these markers among populations, we visualized that the population with greatest difference from the NAT population was the Europeans, with a total of seven markers, all with high statistical significance (rs4883537, rs4883538, rs4883543, rs4883544, rs5744761, and rs4883613 in the *POLE* gene; rs3087377 in the *POLG* gene). On the other hand, the population that differed least from the Amazonian Indigenous was the African population, with only rs11573344, present in POLA1 (*p*–Value = 0.0134). It is worth noting that all variants described here show modifier or moderate impact, according to the prediction of the SNPeff software, which can signify an important phenotypic impact in the individual with these variants. Supplementary Table S1 shows the same analysis as in Table 3 performed on the markers that were not statistically significant.

**Figure 1:**
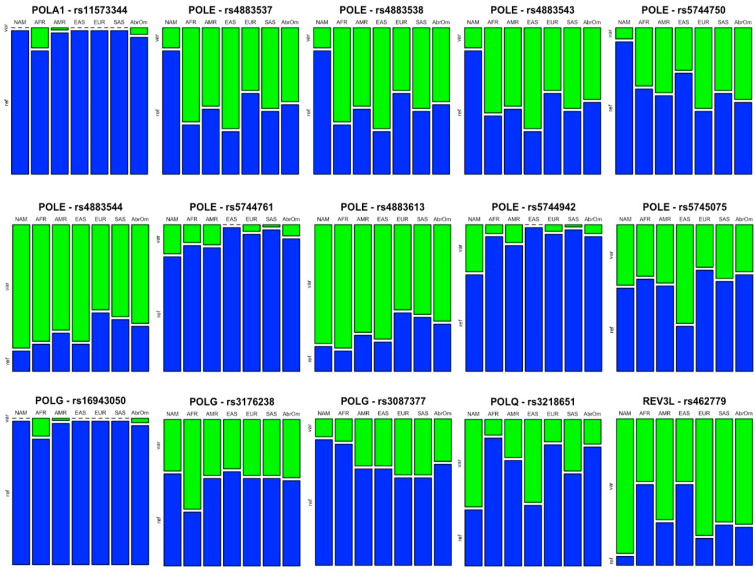
Wild-type and mutant genotype Mosaic plot for each variant with statistical significance in the comparison analysis between the seven study populations (AFR, AMR, EAS, EUR, SAS, NAT and ABraOM).

Figure 1 illustrates only the SNVs that were statistically significant in Table 3. We show a mosaic plot for each variant, with data on the percentage distribution of the wild-type allele (“Reference” or “Ref”) and the mutant allele (“Variant” or “Var”), for each investigated population.

## 4. Discussion

In humans, replication occurs in a coordinated manner with DNA repair mechanisms, which operate in cells to remove or indicate tolerance to DNA damage [28]. DNA polymerases are enzymes that play an important role in both processes, as they function as signaling pathways in cell cycle checkpoint pathways, pointing out the need for a pause in cell division until damaged DNA is repaired and replication is completed [29,30]. Previous studies available in the literature have linked genetic variations in DNA polymerase genes to biological dysfunctions, which are capable of primarily triggering oncogenesis and advancing tumor progression in various tissues [7,31,32,33,34]. The study by Tomasetti et al. demonstrated that, for some tumor types, 77% of mutations in cancer driver genes can be attributed to errors during DNA replication; which can inhibit the expression of a gene important for successful replication or inhibit cell death, a well-known cancer hallmark [7,35].

Although it is understood that the replication and repair gene pathways are determinants for good biological functioning, no studies have screened these genes in Brazilian Amerindian populations. In fact, there is a gap in the knowledge of their genetic and epidemiological component, which is noteworthy, since there are approximately 355,000 Amerindians in 383 demarcated lands in the country [21]. Studies have shown that the settlement of the Indigenous populations was important in determining the number of gene lineages and founder haplotypes observed in these populations, as it triggered a population reduction [36]. Therefore, the genetic structure of contemporary Indigenous populations is relevant to the distribution of complex diseases and rare Mendelian diseases because most communities constitute relatively small and semi-independent gene pools [36]. It is also known that some deleterious mutations are shared among Amerindian populations, either by virtue of a common founding history or by a more recent genetic exchange [36,37,38].

In addition, the lack of genomic knowledge about Brazilian Indigenous populations negatively impacts the knowledge accumulated about the Brazilian population itself, since it has a high degree of interethnic admixture with these Indigenous groups [39,40,41]. Recent studies have shown that a high degree of Amerindian genomic ancestry is associated with the modulation of predisposition and therapeutic response in certain pathologies [25,42,43,44].

Thus, we carried out the characterization of the molecular profile of *POLA1*, *POLE*, *POLG*, *POLQ* and *REV3L* genes, involved in DNA replication and repair pathways in Amerindian populations from the Brazilian Amazon, comparing these findings with genomic data from five continental populations described in the gnomAD database, and with data from the Brazilian population described in the ABraOM database.

Our data demonstrated that the Amerindian populations investigated present variants with allele frequencies that diverge not only from the five continental populations, but also from the population of southeastern Brazil, reinforcing the fact that such groups present a low genetic variability, so that studies with data generated for other world populations may not be extrapolated or applicable to them [26,44,45].

A study published by our research group addressing north Brazilian Amerindian populations and DNA repair genes revealed nine new variants unique to this population, among which some had predicted high and modifier clinical impact [26]. In addition, this study demonstrated that the variants already described had high allele frequency in the Indigenous population, seven of which were associated with modulation in disease predisposition or therapeutic response, demonstrating that knowledge of different patterns in human genetic diversity is important in many areas of medical genetics, and it can be used as a tool to maximize the understanding of susceptibility, diagnosis, prognosis, and therapeutic management for Indigenous populations [26].

In this present study, we also found new variants never described in other populations, possibly unique to NAT populations. Among them, three had clinical impact predicted as modifier by SNPeff software (*POLA1*, *POLE* and *REV3L* genes) and one INDEL type had high clinical impact, present in the POLQ (Polymerase θ) gene, involved in translesion DNA synthesis (TLS), an important pathway that contributes to cell survival by bypassing DNA lesions that have not been repaired by other processes [12,34,46,47]. TLS is performed by seven polymerases that are recruited to the stalled replication forks, allowing damaged cells to complete genome replication, modulating the clinical impact caused by DNA errors in the body. Among the polymerases involved, we find *POLQ* and *REV3L*, investigated here [12,34].

Our results also showed that the modifier variants rs4883544 and rs4883613 of the *POLE* gene were significantly different regarding their distribution in the NAT population when compared to EUR, SAS and ABraOM. DNA POLE (Polymerase ε) is an essential enzyme for successful replication to occur, and also has a 3′–5′ exonuclease activity, which corrects errors in DNA synthesis and helps maintain genomic stability [48]. Genome sequencing applied to cancer patients demonstrates that 3% of colorectal cancers and 7% of endometrial cancers contain mutations involving the exonuclease domain of POLE and are associated with high levels of single-nucleotide polymorphisms (SNPs) in this gene [10,11,49].

Finally, rs3087377 of the *POLG* gene was the SNV that stood out the most among our results, as it was differentially distributed in the NAT population when compared to four of the five continental populations investigated (*p*–Value AMR = 0.0365, *p*–Value EAS = 0.0366, *p*–Value EUR = 0.0004, *p*–Value SAS = 0.0006), as well as when compared to the Brazilian population described in the ABraOM (*p*–Value = 0.058). POLG (Polymerase γ) is considered the replicative as well as the mitochondrial DNA repair polymerase [50]. Different mutations in the POLG gene result in a wide variety of clinical phenotypes such as seizures, neurodegenerative disorders, and liver dysfunction [51,52,53].

This is the first study to characterize the molecular profile of *POLA1*, *POLE*, *POLG*, *POLQ* and *REV3L* genes in Amerindian populations from the Brazilian Amazon, a population whose genetic component has not yet been described in any database of human genetic variability. Molecular epidemiology studies such as this are the basis for inferences about human evolutionary history, so the lack of such data creates a gap in the understanding of several processes investigated by population genetics [26,54,55]. Understanding the genetic variability of Amerindians in genomic studies can aid the investigation of important molecular markers for clinical practice, and it can also generate a great scientific and public health impact for these people and admixed populations with this ethnic group, such as the Brazilian population in general. Thus, we also aim to collaborate with the creation of public policies able to optimize the quality of life of the Amerindian populations from the Brazilian Amazon.

Despite the importance of our data, new studies should be carried out in order to broaden the understanding of the genetic profile of these populations. We also suggest case–control studies to validate the investigations performed in Amerindian populations or uncover new insights regarding the clinical impact of the genetic variations described here.

## 5. Conclusions

Ethnic groups located in the Brazilian Amazon have differential distribution of important molecular markers present in DNA replication and repair pathways. Our findings may contribute to future studies of association between complex diseases in these populations and in the Brazilian population itself. The Amerindian populations investigated present a unique genetic profile, with genetic variants never previously described in other populations. We hope that these data can help establish public policies for this group and for populations admixed with them, such as the Brazilian population.

## Figures and Tables

**Table 1:** Allele frequencies of the variants investigated in the Indigenous population (NAT), in the Brazilian population of Sao Paulo (ABraOM) and in African (AFR), European (EUR), American (AMR), East Asian (EAS) and South Asian (SAS) from the gnomAD database.

Chrom	Position	Reference	Variant	Gene	dbSNP	Impact	Var Type	AFR	AMR	EAS	EUR	SAS	NAT	AbraOM
ChrX	24724557	C	T	*POLA1*	rs11573344	MODIFIER	SNV	0.1381	0.01883	0	0.0006953	0.001091	0	0.044
chr12	132624861	CCAAGG	C	*POLE*	rs375041812	MODIFIER	INDEL	0.1356	0.1887	0.1033	0.1508	0.06555	0.0323	0.142
chr12	132687239	G	A	*POLE*	rs2075784	MODIFIER	SNV	0.2492	0.4231	0.2953	0.5394	0.4381	0.4138	0.453
chr12	132649610	G	A	*POLE*	rs4883537	MODIFIER	SNV	0.6508	0.5484	0.7006	0.4344	0.558	0.1379	0.509
chr12	132624704	C	T	*POLE*	rs1060500879	MODERATE	SNV	0	0	0	0	0	0	ND *
chr12	132648860	G	T	*POLE*	rs4883538	MODIFIER	SNV	0.6617	0.5493	0.7002	0.4335	0.558	0.1379	0.51
chr12	132661490	T	C	*POLE*	rs5744844	MODIFIER	SNV	0.6302	0.5468	0.7003	0.4339	0.5586	0.5405	0.503
chr12	132625418	C	T	*POLE*	rs4883543	MODIFIER	SNV	0.5903	0.5433	0.7009	0.4365	0.5617	0.1379	0.496
chr12	132677497	T	C	*POLE*	rs5744750	MODIFIER	SNV	0.4105	0.4572	0.3003	0.5657	0.4411	0.0833	0.507
chr12	132677409	G	A	*POLE*	rs5744751	MODERATE	SNV	0.0178	0.0542	0.102	0.1097	0.0576	0.0086	0.069
chr12	132625361	T	C	*POLE*	rs4883544	MODIFIER	SNV	0.8083	0.7394	0.8047	0.5885	0.6356	0.8594	0.685
chr12	132673431	C	A	*POLE*	rs5744776	MODIFIER	SNV	0.01785	0.05457	0.1029	0.1096	0.05733	0	0.069
chr12	132642138	C	T	*POLE*	rs5744951	MODIFIER	SNV	0.6612	0.5492	0.701	0.4338	0.5574	0.5	0.512
chr12	132675353	G	A	*POLE*	rs5744761	MODIFIER	SNV	0.1186	0.1351	0.0005	0.04453	0.01076	0.1957	0.077
chr12	132649573	T	C	*POLE*	rs4883613	MODIFIER	SNV	0.8576	0.745	0.8037	0.5887	0.6267	0.8243	0.667
chr12	132643363	G	A	*POLE*	rs5744942	MODIFIER	SNV	0.06067	0.127	0.0006	0.04441	0.0099	0.3261	0.063
chr12	132625629	G	A	*POLE*	rs5745075	MODIFIER	SNV	0.3656	0.4121	0.6927	0.304	0.374	0.4167	0.322
chr12	132624855	AGGAG	A	*POLE*	rs199947622	MODIFIER	INDEL	0.1356	0.1887	0.1033	0.1508	0.06581	0.0323	0.142
chr12	132673532	C	T	*POLE*	rs4883555	MODIFIER	SNV	0.6301	0.5469	0.6992	0.4343	0.5574	0.1207	0.502
chr15	89321383	G	A	*POLG*	rs16943050	MODIFIER	SNV	0.1293	0.02243	0	0.001367	0.00062	0	0.036
chr15	89325718	G	A	*POLG*	rs3176190	MODIFIER	SNV	0.2214	0.02926	0.0006	0.00195	0.0008	0	0.054
chr15	89319135	T	C	*POLG*	rs2246900	MODIFIER	SNV	0.1521	0.3283	0.3248	0.3948	0.3899	0.25	0.296
chr15	89323923	A	G	*POLG*	rs2072267	MODIFIER	SNV	0.7859	0.4702	0.3889	0.4712	0.4805	0.2857	0.509
chr15	89317196	C	T	*POLG*	rs3176238	MODIFIER	SNV	0.6216	0.3954	0.3396	0.3969	0.3923	0.3529	0.409
chr15	89321085	G	GCTAC	*POLG*	rs2307433	MODIFIER	INDEL	0.791	0.4503	0.4316	0.4692	0.4868	0.2778	0.512
chr15	89316763	C	A	*POLG*	rs3087374	MODERATE	SNV	0.0148	0.0458	0.0002	0.0843	0.01863	0.0079	0.048
chr15	89327116	G	A	*POLG*	rs2307453	MODIFIER	SNV	0.0637	0.0053		0.0002	0.0004	0	0.012
chr15	89317555	A	C	*POLG*	rs2307438	MODIFIER	SNV	0.5184	0.4216	0.3537	0.3987	0.3904	0	0.391
chr15	89319110	A	G	*POLG*	rs2302084	MODIFIER	SNV	0.1775	0.3323	0.3237	0.3962	0.3912	0.246	0.3
chr15	89316701	A	AC	*POLG*	rs3087377	MODIFIER	INDEL	0.1517	0.3254	0.3221	0.3927	0.3876	0.119	0.295
chr15	89324193	C	T	*POLG*	rs2307450	MODERATE	SNV	0.0007	0.0497	0	0.0005	0	0.1172	0.016
chr3	121490047	T	C	*POLQ*	rs3218636	MODERATE	SNV	0.01476	0.1662	0.0011	0.02929	0.01117	0.5164	0.057
chr3	121544873	C	A	*POLQ*	rs702017	MODERATE	SNV	0.9628	0.9973	1	0.9999	0.9998	1	0.994
chr3	121533021	A	C	*POLQ*	rs55748151	MODERATE	SNV	0.0011	0.0229	0.0886	0.0052	0.0046	0	0.009
chr3	121489329	T	C	*POLQ*	rs3218651	MODERATE	SNV	0.1148	0.2704	0.5704	0.1552	0.3618	0.6129	0.165
chr3	121436127	T	C	*POLQ*	rs1381057	MODERATE	SNV	0.653	0.7111	0.8898	0.6827	0.8662	0.75	0.689
chr3	121489764	G	A	*POLQ*	rs34778629	MODERATE	SNV	0.0587	0.0074	0	0.0005	0.0004	0	0.013
chr3	121544797	C	A	*POLQ*	rs61757738	MODERATE	SNV	0.001	0.01126	0.0004	0.0015	0.0023	0.0156	0.003
chr3	121489401	G	T	*POLQ*	rs745401535	HIGH	SNV	ND *	ND *	ND *	ND *	ND *	0.009	0.001
chr3	121489986	G	C	*POLQ*	rs3218649	MODERATE	SNV	0.5618	0.6551	0.8884	0.6318	0.8239	0.7295	0.618
chr6	111390029	C	CG	*REV3L*	rs11376056	MODIFIER	INDEL	0.4335	0.6881	0.4665	0.8132	0	0.8667	0.737
chr6	111374684	G	A	*REV3L*	rs462779	MODERATE	SNV	0.4334	0.7095	0.4448	0.8131	0.717	0.9344	0.732
chr6	111375096	G	A	*REV3L*	rs1284920600	MODERATE	SNV	0	0.00002	0	0	0	0	ND *
chr6	111367879	C	T	*REV3L*	rs3218606	MODERATE	INDEL	0.00287	0.06022	0.02637	0.01154	0.01329	0.0391	0.022
chr6	111374888	T	C	*REV3L*	rs458017	MODERATE	SNV	0.04429	0.04761	0.0019	0.06335	0.04453	0	0.052
chr6	111372984	G	A	*REV3L*	rs17539651	MODERATE	SNV	0.1233	0.01565	0	0.00026	0.0003	0.0082	0.026

* ND = No data.

**Table 2:** New variants found in the Indigenous population from the Brazilian Amazon.

Gene	Chrom	Position	Region Detailed	Reference	Variant	Impact	Var Type	Change Protein	Alteration
*POLA1*	ChrX	24741992	INTRON	C	A	MODIFIER	SNV	c.2329–10C > A	-
*POLA1*	ChrX	24724348	NON-SYNONYMOUS CODING	A	G	MODERATE	SNV	p.Asn399Ser	MISSENSE
*POLE*	chr12	132687424	UTR_5_PRIME	T	C	MODIFIER	SNV	c.-109A > G	-
*POLG*	chr15	89333217	NON-SYNONYMOUS CODING	G	A	MODERATE	SNV	p.Pro180Ser	MISSENSE
*POLQ*	chr3	121473365	FRAME SHIFT	CTG	C	HIGH	INDEL	p.Gln2176fs	FRAME SHIFT
*POLQ*	chr3	121436148	NON-SYNONYMOUS CODING	T	C	MODERATE	SNV	p.Glu2506Gly	MISSENSE
*POLQ*	chr3	121488382	NON-SYNONYMOUS CODING	C	A	MODERATE	SNV	p.Val1517Leu	MISSENSE
*REV3L*	chr6	111365457	INTRON	T	C	MODIFIER	SNV	c.6674–113A > G	-

**Table 3:** Pairwise comparison (*p*-value) of allelic frequencies in Amerindians (NAT), the Brazilian population described in ABraOM and continental populations (African (AFR), American (AMR), East Asian (EAS), European (EUR) and South Asian (SAS) populations) described in the gnomAD database.

Gene	SNPID	Var Type	Impact	AFR	AMR	EAS	EUR	SAS	ABraOM
*POLA1*	rs11573344	SNV	MODIFIER	0.0134	1	1	1	1	1
*POLE*	rs4883537	SNV	MODIFIER	0	0	0	0.0001	0	0
*POLE*	rs4883538	SNV	MODIFIER	0	0	0	0.0001	0	0
*POLE*	rs4883543	SNV	MODIFIER	0	0	0	0.0001	0	0
*POLE*	rs5744750	SNV	MODIFIER	0	0	0.0024	0	0	0
*POLE*	rs4883544	SNV	MODIFIER	1	1	1	0.0004	0.0107	0.134
*POLE*	rs5744761	SNV	MODIFIER	1	1	0	0.0004	0	0.219
*POLE*	rs4883613	SNV	MODIFIER	1	1	1	0.0054	0.0585	0.261
*POLE*	rs5744942	SNV	MODIFIER	0	0.0024	0	0	0	0
*POLE*	rs5745075	SNV	MODIFIER	1	1	0.0008	1	1	1
*POLG*	rs3176238	SNV	MODIFIER	0.0023	1	1	1	1	1
*POLG*	rs16943050	SNV	MODIFIER	0.0214	1	1	1	1	1
*POLG*	rs3087377	INDEL	MODIFIER	1	0.0365	0.0366	0.0004	0.0006	0.058
*POLQ*	rs3218651	SNV	MODERATE	0	0	0.857	0	0.0062	0
*POLQ*	rs3218649	SNV	MODERATE	0.473	1	0.0478	1	1	1
*REV3L*	rs462779	SNV	MODERATE	0	0.001	0	0.8278	0.0016	0.007

## Data Availability

The authors confirm that the data supporting the findings of this study are available within the article and its Supplementary Material. Raw data of the studied genes are available from the corresponding author, upon reasonable request.

## Supplementary Materials

The following supporting information can be downloaded at: https://www.mdpi.com/article/10.3390/genes14010053/s1, Table S1: Description of non-significant results of comparison between the allele frequency of Amerindian populations (NAT), the Brazilian population described in ABraOM and continental populations (African (AFR), American (AMR), East Asian (EAS), European (EUR) and South asian (SAS)) described in the gnomAD database.
