# Supplementary material for: Characterization of DNA Polymerase Genes in Amazonian Amerindian Populations
Source: Genes (Basel). 2022 Dec 24;14(1):53. doi: 10.3390/genes14010053 (PMC9859017; doi:10.3390/genes14010053)
Supplement: Supplementary file 1 [file genes-14-00053-s001.zip › genes-2022343-supplementary.pdf]

Table S1. Description of non-significant results of comparison between the allele frequency of Amerindian populations (NAT), the Brazilian population described in ABraOM and continental populations (African (AFR), American (AMR), East Asian (EAS), European (EUR) and South asian (SAS))) described in the gnomAD database.

Gene	SNPID	Variation Type	Impact	African	American	East Asian	European	South Asian	ABraOM
POLE	rs375041812	INDEL	MODIFIER	0.8464	0.029	1	0.3928	1	1
POLE	rs2075784	SNV	MODIFIER	0.509	1	1	1	1	1
POLE	rs1060500879	SNV	MODERATE	1	1	1	1	1	1
POLE	rs5744844	SNV	MODIFIER	1	1	0.8407	1	1	1
POLE	rs5744751	SNV	MODERATE	1	1	1	0.7724	1	1
POLE	rs5744776	SNV	MODIFIER	1	1	0.1372	0.0895	1	0.119
POLE	rs5744951	SNV	MODIFIER	0.7212	1	0.0786	1	1	1
POLE	rs199947622	INDEL	MODIFIER	0.8464	0.029	1	0.3928	1	1
POLE	rs4883555	SNV	MODIFIER	0	0	0	0	0	0
POLG	rs3176190	SNV	MODIFIER	0	1	1	1	1	1
POLG	rs2246900	SNV	MODIFIER	1	1	1	1	1	1
POLG	rs2072267	SNV	MODIFIER	0	0.2206	1	0.2193	0.1395	0.06
POLG	rs3176238	INDEL	MODIFIER	0	0.6974	1	0.2252	0.0912	1
POLG	rs3087374	SNV	MODERATE	1	1	1	1	1	1
POLG	rs2307453	SNV	MODIFIER	1	1	ND	ND	1	1
POLG	rs2307438	SNV	MODIFIER	0	0	0	0	0	0
POLG	rs2302084	SNV	MODIFIER	1	1	1	1	1	1
POLG	rs2307450	SNV	MODERATE	0	1	0	0	0	0.065
POLQ	rs3218636	SNV	MODERATE	0	0	0	0	0	0
POLQ	rs702017	SNV	MODERATE	1	1	1	1	1	1
POLQ	rs55748151	SNV	MODERATE	1	1	0.5396	1	1	1
POLQ	rs1381057	SNV	MODERATE	1	1	0.1685	1	1	1

POLQ	rs34778629	SNV	MODERATE	1	1	1	1	1	1
POLQ	rs61757738	SNV	MODERATE	1	1	1	1	1	1
POLQ	rs745401535	SNV	HIGH	ND*	ND*	ND*	ND*	1	1
REV3L	rs11376056	INDEL	MODIFIER	0	0.2352	0	1	0	0.123
REV3L	rs1284920600	SNV	MODERATE	1	1	1	1	1	1
REV3L	rs3218606	SNV	MODERATE	1	1	1	1	1	1
REV3L	rs458017	SNV	MODERATE	1	1	1	1	1	1
REV3L	rs17539651	SNV	MODERATE	0.3271	1	0.622	1	1	1
